# Social Media Use and Personal Relative Deprivation Among Urban Residents in China: A Moderated Mediation Model

**DOI:** 10.3390/bs15070962

**Published:** 2025-07-16

**Authors:** Yihua Liu, Xiaoge Zhao

**Affiliations:** 1 Department of Sociology and Psychology, School of Public Administration, Sichuan University, Chengdu 610065, China; yihualiu@stu.scu.edu.cn; 2 HNU-ASU International College, Hainan University, Haikou 570228, China

**Keywords:** personal relative deprivation, social media use, subjective social status, belief in a just world, urban residents in China

## Abstract

Personal relative deprivation (PRD) is closely linked to a range of mental health problems. In the digital era, the association between social media use and PRD has received increasing attention. However, most studies have been conducted in Western contexts, and the underlying mechanisms in China remain unclear. This study examined the relationship between social media use and PRD among 2504 adult urban residents in China. Based on relative deprivation theory, it further explored the mediating role of subjective social status and the moderating role of belief in a just world. Results revealed that social media use was negatively associated with PRD. Subjective social status mediated this relationship: social media use was positively associated with subjective social status, while subjective social status was negatively associated with PRD. Moreover, belief in a just world strengthened the direct negative link between social media use and PRD, as well as the positive link between social media use and subjective social status. These findings suggest that social media are not always a risk factor for mental health. Their impact should be considered within specific cultural contexts and regulatory policies.

## 1. Introduction

Personal relative deprivation (PRD) refers to the judgment that one is disadvantaged compared to others, often accompanied by feelings of anger and resentment (Pettigrew, 2016). It has been closely linked to a range of mental health problems. For example, Beshai et al. (2017) found a significant positive association between PRD and depressive symptoms in a study of 2999 adults from English-speaking countries. Similarly, Nadler et al. (2020) reported a significant positive link between PRD and generalized anxiety disorder. In China, several studies found a significant positive association between PRD and suicidal ideation (J. Zhang & Tao, 2013; Zhao et al., 2024). Even after controlling for all covariates, individuals who experienced PRD were over four times more likely to report suicidal risk than those who did not. These findings suggest that PRD undermines mental health and should be considered a critical issue in public mental health.

Relative deprivation theory provides a framework for understanding the development of PRD (Crosby, 1976; Smith et al., 2012). According to the theory, social comparison is a necessary condition for PRD to emerge. Individuals who perceive themselves as “deserving but deprived” during the comparison process tend to experience negative emotions. Pettigrew (2016) further emphasized that PRD arises from individuals’ subjective perception of disadvantage in comparison with others. In the era dominated by traditional media, comparison targets were typically limited to close social groups, such as neighbors and friends. However, with the rise of digital technologies, these targets have expanded. In particular, the widespread use of social media allows individuals to compare themselves across geographic and socioeconomic boundaries. People are now exposed not only to the hardships of lower-status groups but also to the conspicuous displays of higher-status individuals. As a result, PRD may be alleviated through favorable downward comparisons or intensified through unfavorable upward comparisons.

According to official data from China, internet penetration in urban areas reached 85.3% by the end of 2024 (China Internet Network Information Center, 2025). Social media platforms such as WeChat and Weibo have become primary sources of information for urban residents. As a commonly used tool in daily life, social media may play a crucial role in shaping individuals’ social perceptions (Scardigno et al., 2024). However, research on the relationship between social media use and PRD among urban residents in China remains limited. This gap hinders the identification and intervention of relevant mental health problems. Based on relative deprivation theory, the present study explores the association between social media use and PRD among Chinese urban residents. The findings aim to provide empirical evidence to support mental health interventions in this population.

### 1.1. Social Media Use and PRD

Research on the relationship between social media use and PRD remains inconclusive. Some research suggests that exposure to others’ positive self-presentation on social media often triggers upward social comparisons, thereby increasing individuals’ PRD (Kim & Chock, 2015; Park & Park, 2024). In contrast, another study highlights the potential benefits of social media. It suggests that social media expand social networks and enhances social capital, which may reduce individuals’ PRD (Cho, 2014). These divergent findings largely stem from differing theoretical perspectives. Some studies adopt impression management and social comparison theory, while others draw on social capital theory. Although each provides partial insight, neither offers a comprehensive framework for understanding the psychological mechanisms linking social media use to PRD. Moreover, recent research suggests that social media use may not be consistently associated with negative outcomes. For example, Goh et al. (2025) found no clear association between social media use and body image dissatisfaction. Similarly, Lilly et al. (2023) reported no significant link between social media use and PRD among adults in New Zealand, indicating that such use does not necessarily increase feelings of deprivation. However, most of these findings are based on samples from Western countries. Their applicability to the Chinese context remains uncertain. In China, stricter content regulation on social media may shape a distinct information environment, particularly in how reference groups and social values are presented (Al-Zaman, 2024). Therefore, further research is needed to adopt a more integrative framework to examine the relationship between social media use and PRD in the Chinese context.

According to relative deprivation theory, PRD arises not from a lack of material resources but from perceived comparative disadvantage in social comparison, particularly when this disadvantage is attributed to external injustice (Crosby, 1976; Smith et al., 2012). In China, the urban–rural development is highly unbalanced (J. Liu et al., 2017). Compared to rural residents, urban residents generally have better access to education, employment, and public services. As a result, they are structurally advantaged, even when making comparisons on social media. Meanwhile, the Chinese government has intensified regulation of social media content (Cyberspace Administration of China, 2024). Ostentatious displays of wealth and extravagant lifestyles are discouraged, while narratives that promote hard work and upward mobility are emphasized. These external conditions reduce the likelihood of encountering content that triggers upward comparisons, making urban residents less likely to feel disadvantaged. In addition, from an internal psychological perspective, research suggests that upward comparisons can be psychologically threatening and are often avoided, particularly when individuals feel vulnerable (Brickman & Bulman, 1977). In contrast, downward comparison theory posits that people are more likely to compare themselves with others who are worse off in order to protect self-esteem and maintain subjective well-being (Wills, 1981). This self-protective strategy is especially relevant in the context of Chinese collectivist culture, where preserving emotional harmony and maintaining face are highly valued. Furthermore, influenced by Confucian values, Chinese individuals tend to attribute life outcomes to personal effort rather than systemic unfairness (Ge & Hou, 2022). When urban residents encounter others’ success online, they are more likely to interpret it as a result of diligence rather than privilege. This attribution style may foster self-motivation instead of perceived injustice, thereby weakening the cognitive foundation necessary for PRD to arise. Additionally, social media use may increase social capital by broadening social networks and improving access to supportive resources (Cho, 2014). These effects can enhance individuals’ sense of self-worth (Zhai, 2021), further alleviating PRD. Taken together, in China’s unique cultural and regulatory context, social media use may reduce PRD rather than increase it. Therefore, we propose the following context-specific hypothesis:

**Hypothesis 1.**  *Under China’s distinct cultural values and content regulation, social media use is negatively associated with PRD among urban residents.*

### 1.2. Subjective Social Status as a Mediator

Relative deprivation theory highlights that a key condition for the emergence of PRD is the perception of comparative disadvantage (Crosby, 1976; Smith et al., 2012). This perception is often related to the subjective evaluation of one’s own social position. Subjective social status refers to an individual’s subjective assessment of their position within the social structure (Adler et al., 2000). Compared to objective socioeconomic indicators such as income, occupation, or education, subjective social status more accurately reflects the psychological experiences in the social comparison process. Social comparison theory suggests that, in the absence of absolute reference standards, individuals often rely on information from others to construct their own social positioning (Festinger, 1954). Social media significantly broaden the scope of comparison, allowing individuals to across class boundaries and connect with a wider range of social groups, thus influencing their understanding of their social status. On the one hand, social media facilitate the acquisition of skills and knowledge, which can increase social, cultural, and economic capital, boosting individuals’ confidence in upward mobility (Eynon et al., 2018). On the other hand, social media provide a platform for social interaction, which enhances self-esteem and self-identity (Marengo et al., 2021; Wang et al., 2021). A national survey in China has found a significant positive association between social media use and subjective social status (J. Zhang et al., 2024). The social networks constructed through social media not only serve as actual resources for upward mobility but also provide emotional support, improving individuals’ positive evaluations of their social position on a cognitive level. Furthermore, Callan et al. (2017) indicated that individuals with higher subjective social status typically experience lower levels of PRD. Such individuals face fewer upward comparisons in social comparison processes and engage in comparisons less frequently, making them less likely to experience PRD overall. Other studies also revealed a significant negative association between subjective social status and PRD (Kraft & Kraft, 2023). This suggests that subjective social status may serve as a mediator in the relationship between social media use and PRD. Therefore, the following hypotheses are proposed:

**Hypothesis 2.**  *Subjective social status mediates the relationship between social media use and PRD.*

**Hypothesis 2a.**  *Social media use is positively associated with subjective social status.*

**Hypothesis 2b.**  *Subjective social status is negatively associated with PRD.*

### 1.3. Belief in a Just World as a Moderator

Relative deprivation theory further posits that the emergence of PRD depends not only on individuals’ perception of being in a comparative disadvantaged position but also on whether they attribute this disadvantage to social injustice (Crosby, 1976; Smith et al., 2012). The belief in a just world refers to the assumption that the world is fundamentally fair and that people generally get what they deserve (Lerner & Miller, 1978). Belief in a just world influences how individuals respond to inequality, both emotionally and cognitively. Emotionally, it serves as a buffer against the distress caused by upward social comparisons. Individuals with a strong belief in a just world are more likely to attribute wealth and poverty to personal causes rather than structural inequality, in order to preserve their belief that the world is fundamentally fair (Furnham, 2003; Hafer & Bègue, 2005). During upward comparisons, this attribution style helps reduce negative emotions such as envy and resentment, which are key emotional triggers of PRD (Feather, 2015; Pettigrew, 2016). Research also shows that individuals with stronger belief in a just world exhibit greater psychological resilience, better anger regulation, and lower levels of neuroticism (Bartholomaeus et al., 2024; Dalbert & Filke, 2007; Nudelman, 2013). This belief is further associated with favorable physical health outcomes, including reduced inflammation and improved sleep quality (Levine et al., 2017). These psychological and physiological resources help individuals remain emotionally stable in the face of social comparison. Cognitively, belief in a just world also shapes how people interpret social hierarchies. Those with strong belief in a just world are more likely to view success as fair and attainable. When observing others’ achievements on social media, they are more likely to feel inspired rather than threatened. This fosters a sense of personal control, strengthens confidence in upward mobility through effort, and enhances positive evaluations of one’s own social status (Furnham, 2003; Kiral Ucar et al., 2019). In contrast, individuals with weaker belief in a just world are more likely to interpret social success as a result of structural unfairness. This perception may intensify feelings of deprivation and lower perceived social standing. Recent research supports this mechanism. For example, Pan and Zhao (2024) found that individuals with lower levels of belief in a just world tended to report higher levels of PRD. Taken together, belief in a just world may moderate both the emotional and cognitive effects of social media use. It can reduce the psychological burden of social comparison and help individuals maintain a more positive perception of their social position. Therefore, the following hypotheses are proposed:

**Hypothesis 3.**  *Belief in a just world strengthens the direct negative association between social media use and PRD.*

**Hypothesis 4.**  *Belief in a just world strengthens the positive association between social media use and subjective social status.*

### 1.4. Present Study

**Figure 1:**
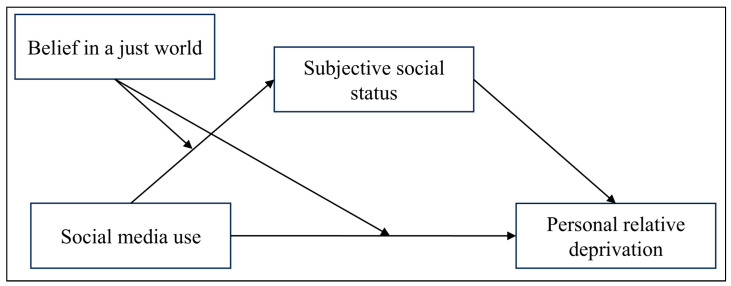
Moderated mediation model of the present study.

Previous research on the relationship between social media use and PRD has focused primarily on Western populations (Lilly et al., 2023; Park & Park, 2024). The present study proposes a moderated mediation model (see Figure 1) to examine this association among urban residents in China. It further explores the mediating role of subjective social status and the moderating role of belief in a just world.

## 2. Materials and Methods

### 2.1. Participants and Procedures

This cross-sectional study was conducted from January to March 2025. Participants were recruited from the sample pool of the online survey platform Wenjuanxing (https://www.wjx.cn). This sample pool covers all provincial-level regions in mainland China and has been widely used in nationwide survey studies (Ning et al., 2020; Yu et al., 2021). Based on data from the Seventh National Population Census, we set quota ranges for age and gender to approximate the key demographic characteristics of urban residents in China. The inclusion criteria were being an adult urban resident and a social media user. To prevent duplicate responses, each IP address was allowed to submit the questionnaire only once. Anonymous self-administered questionnaires were used. Before beginning the survey, all participants read and signed an electronic informed consent form. Upon completion, they received a monetary reward of CNY 5 (approximately USD 0.7). A total of 2717 participants completed the survey. To ensure data quality, we included an attention check item (e.g., “Please select ‘strongly disagree’ for this item”). Participants who failed this check were excluded. We also conducted consistency checks by examining response patterns on reverse-coded items and calculating response times. Questionnaires completed in less than 3 min or showing inconsistent answers on logically paired items were removed. After excluding 213 responses that failed quality control checks, 2504 valid questionnaires were retained. The final valid response rate was 92.16%.

The sociodemographic characteristics of the participants are presented in Table 1. Specifically, 38.14% of participants were aged 18 to 35 years, 52.76% were aged 36 to 59 years, and 9.11% were aged 60 years or older. In terms of gender, 47.84% were male and 52.16% were female. Regarding marital status, 76.00% of participants were married and 24.00% were unmarried. For education level, 6.51% had completed primary school or below, 51.64% had completed middle school, and 41.85% had attained a college degree or higher. Concerning annual family income, 22.16% earned CNY 60,000 or less, 35.58% earned between CNY 60,001 and CNY 150,000, and 42.45% earned more than CNY 150,001. Additionally, 76.68% of participants had either medical or pension insurance, while 23.72% did not hold such insurance.

### 2.2. Measures

#### 2.2.1. Social Media Use

The Social Media Use Integration Scale (SMUIS) was used to measure social media use (Jenkins-Guarnieri et al., 2013). In this study, the term “Facebook” in the original scale was replaced with “WeChat/Weibo” to better fit the Chinese context. The SMUIS captures not only the frequency of social media use but also how individuals integrate social media into their daily lives. It is a comprehensive tool applicable to various social media platforms. The scale has demonstrated good reliability in Chinese populations (Guo et al., 2024). It consists of 10 items (e.g., “I enjoy checking my WeChat/Weibo account”) rated on a 6-point Likert scale (1 = strongly disagree, 6 = strongly agree). Higher scores indicate greater levels of social media use. In this study, Cronbach’s α for the scale was 0.748.

#### 2.2.2. Personal Relative Deprivation

The Personal Relative Deprivation Scale (PRDS) was used to assess personal relative deprivation (Callan et al., 2011). The PRDS has shown good reliability in Chinese samples (H. Zhang et al., 2024). It includes 10 items (e.g., “I feel deprived when I think about what I have compared to what other people like me have”) rated on a 6-point Likert scale (1 = strongly disagree, 6 = strongly agree). Higher scores reflect higher levels of personal relative deprivation. In this study, Cronbach’s α for the scale was 0.843.

#### 2.2.3. Subjective Social Status

Subjective social status was measured using an adapted 5-point version of the MacArthur Scale (Adler et al., 2000), where participants rated their perceived position in society from 1 (lowest) to 5 (highest). Although the original scale uses a 10-rung ladder to capture finer distinctions, the 5-point version is widely used in Chinese research for its simplicity and cultural relevance. It has been adopted in authoritative large-scale surveys such as the China Family Panel Studies (Zou et al., 2020). Moreover, research suggests that data from the 10-rung scale are often collapsed into five categories without significant information loss, indicating comparable sensitivity (Chen & Fan, 2015). In diverse urban populations, the simplified format also helps reduce respondent burden and improve data quality.

#### 2.2.4. Belief in a Just World

The Belief in a Just World Scale (BJWS) was used to assess belief in a just world (Dalbert, 1999). The BJWS has demonstrated good reliability among Chinese populations (Xiong & Hu, 2022). It consists of 13 items (e.g., “I think basically the world is a just place”) rated on a 6-point Likert scale (1 = strongly disagree, 6 = strongly agree). Higher scores indicate a stronger belief in a just world. In this study, Cronbach’s α for the scale was 0.852.

### 2.3. Statistical Analysis

Data analysis was conducted using IBM SPSS (version 22.0) and the PROCESS macro (Hayes, 2013). First, Pearson correlation was performed to examine the relationships between social media use, PRD, subjective social status, and belief in a just world. Next, the PROCESS macro was used to test for mediation and moderation effects. Specifically, Model 4 was used to test the mediating role of subjective social status. A 95% confidence interval (CI) based on 5000 bootstrap samples was generated, and a significant mediation effect was concluded if the CI did not include zero (Preacher & Hayes, 2008). Model 8 was used to test the moderating role of belief in a just world. Sociodemographic characteristics (age, gender, marital status, education level, annual family income, and social security status) were controlled as covariates in both the mediation and moderation analyses. All regression coefficients reported in the main text were unstandardized estimates. Standardized coefficients are provided in the Supplementary Material for reference. A *p* value < 0.05 was considered statistically significant.

## 3. Results

### 3.1. Preliminary Analysis

As shown in Table 2, the mean scores (and standard deviations) for social media use, PRD, subjective social status, and belief in a just world were 3.83 (*SD* = 0.98), 3.85 (*SD* = 0.78), 2.39 (*SD* = 0.87), and 4.17 (*SD* = 1.06), respectively. Correlation analysis revealed that social media use was significantly negatively correlated with PRD (*r* = −0.257, *p* < 0.001), and significantly positively correlated with subjective social status (*r* = 0.153, *p* < 0.001) and belief in a just world (*r* = 0.138, *p* < 0.001). PRD was significantly negatively correlated with both subjective social status (*r* = −0.498, *p* < 0.001) and belief in a just world (*r* = −0.193, *p* < 0.001). Subjective social status and belief in a just world were significantly positively correlated (*r* = 0.224, *p* < 0.001).

### 3.2. Mediation Analysis

This study tested whether subjective social status mediated the relationship between social media use and PRD (see Table 3). First, the total effect of social media use on PRD was significant (*B* = −0.117, *p* < 0.001), supporting Hypothesis 1. Additionally, social media use was positively associated with subjective social status (*B* = 0.072, *p* < 0.001), and subjective social status was negatively associated with PRD (*B* = −0.386, *p* < 0.001), supporting Hypotheses 2a and 2b. Further analysis indicated that the direct effect of social media use on PRD remained significant (*B* = −0.089, *p* < 0.001). Bootstrap analysis showed that the indirect effect of social media use on PRD through subjective social status was significant (*B* = −0.028, 95% *CI* = [−0.045, −0.012]), accounting for 23.93% of the total effect. These results support Hypothesis 2, indicating that subjective social status partially mediated the relationship between social media use and PRD.

### 3.3. Moderation Analysis

**Figure 2:**
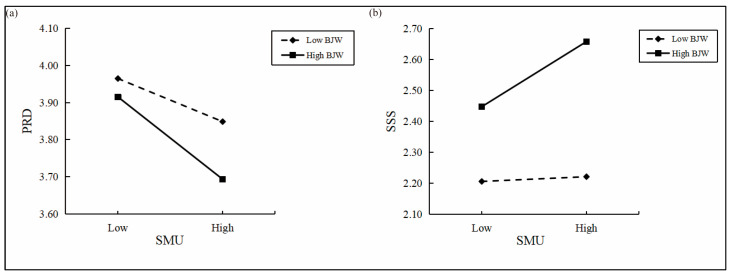
Simple slope analysis. Note: SMU = social media use, PRD = personal relative deprivation, SSS = subjective social status, BJW = belief in a just world. (**a**) Moderating role of BJW between SMU and PRD. (**b**) Moderating role of BJW between SMU and SSS.

This study examined whether belief in a just world moderates the relationship between social media use and PRD (see Table 4). When PRD was the dependent variable, the interaction term between social media use and belief in a just world was significant (*B* = −0.025, *p* < 0.05), indicating a moderating effect. To further illustrate this effect, a simple slope analysis was conducted by dividing participants into high and low belief in a just world groups (±1 *SD* from the mean). As shown in Figure 2, the negative association between social media use and PRD was stronger in the high-belief group (+1 *SD*, *B* = −0.113, *p* < 0.001) and weaker in the low-belief group (−1 *SD*, *B* = −0.059, *p* < 0.01). These findings suggest that higher levels of belief in a just world strengthen the direct negative relationship between social media use and PRD, supporting Hypothesis 3.

In addition, the moderating effect of belief in a just world on the relationship between social media use and subjective social status was also tested. As shown in Table 4, the interaction between social media use and belief in a just world was significant when subjective social status was the dependent variable (*B* = 0.047, *p* < 0.01). A simple slope analysis was again conducted. Results (see Figure 2) indicated that the positive relationship between social media use and subjective social status was significant in the high-belief group (+1 *SD*, *B* = 0.107, *p* < 0.001), but nonsignificant in the low-belief group (−1 *SD*, *B* = 0.008, *p* > 0.05). These results suggest that higher levels of belief in a just world strengthen the positive relationship between social media use and subjective social status, supporting Hypothesis 4.

## 4. Discussion

Based on relative deprivation theory, this study examined the relationship between social media use and PRD among urban residents in China. In addition, we tested the roles of subjective social status and belief in a just world. The findings revealed that social media use was negatively associated with PRD. Subjective social status mediated this relationship. Moreover, belief in a just world moderated both the direct relationship between social media use and PRD, and the relationship between social media use and subjective social status.

### 4.1. Relationship Between Social Media Use and PRD

This study is the first to investigate the relationship between social media use and PRD among urban residents in China. Previous studies have primarily focused on Western countries, with limited evidence from China (Lilly et al., 2023; Park & Park, 2024). In recent decades, China has experienced rapid development, and the internet penetration rate in urban areas has reached 85.3% (China Internet Network Information Center, 2025). Social media platforms such as WeChat and Weibo have become essential tools for information access and social interaction among urban residents. Therefore, examining the psychosocial effects of social media use in the Chinese context holds significant practical value. Our findings revealed a significant negative association between social media use and PRD among Chinese urban residents. According to relative deprivation theory, PRD arises from two key conditions: perceiving oneself to be in a comparatively disadvantaged social position and attributing this disadvantage to external injustice (Crosby, 1976; Smith et al., 2012). Compared with rural residents, urban residents in China possess distinct structural advantages (J. Liu et al., 2017). When using social media, they are less likely to perceive themselves as disadvantaged, especially when comparisons are made with rural populations. Additionally, traditional Chinese culture emphasizes effort-based attributions (Ge & Hou, 2022). Urban residents are more likely to interpret others’ success on social media as the result of hard work rather than systemic privilege. These structural advantages and attribution tendencies together weaken the cognitive and attributional foundations required for PRD to arise. Furthermore, social media use expands individuals’ social networks, facilitating the accumulation of social capital (Ali-Hassan et al., 2015; Ostic et al., 2021). Previous research has shown that social capital is a crucial protective factor for mental health and can effectively buffer negative emotions (Phongsavan et al., 2006; Sotaquirá et al., 2022). In the context of rapid urbanization and growing social mobility, urban residents in China face significant uncertainty and stress. Chinese collectivist culture emphasizes the embeddedness and stability of social relationships (S. S. Liu et al., 2020). Social media help maintain interpersonal bonds in a digital form, enhancing individuals’ sense of belonging and identity (Benson et al., 2019), thereby reducing PRD. It is worth noting that our findings contrast with those from a Korean study. Yang (2020) found a positive relationship between social media use and PRD in the Korean population. This discrepancy may be attributed to the varying levels of content regulation across social media platforms in the two countries (Al-Zaman, 2024). In China, stricter governance of online content limits displays of wealth and excessive consumption, and promotes positive and uplifting values (Cyberspace Administration of China, 2024). This content structure reduces users’ exposure to ostentatious content, which in turn lowers the likelihood of upward social comparison, thereby helping to mitigate PRD. These findings together with other studies suggest that social media are not always a risk factor for mental health (Berryman et al., 2018). Their impact should be considered within specific cultural contexts and regulatory policies. Future research with more rigorous designs, such as longitudinal or experimental approaches, is needed to explore how the positive potential of social media can be leveraged in mental health interventions.

### 4.2. Mediating Role of Subjective Social Status

This study found that subjective social status mediated the relationship between social media use and PRD. Specifically, social media use was positively associated with subjective social status, while subjective social status was negatively associated with PRD. This finding offers a deeper understanding of the mechanisms underlying the development of PRD. According to relative deprivation theory, an important condition for the emergence of PRD is the perception of being in a comparative disadvantaged position during social comparisons (Crosby, 1976; Smith et al., 2012). Subjective social status is precisely an assessment of one’s relative position in the social structure (Adler et al., 2000). On the one hand, individuals can use social media to acquire information and learn skills, which helps to enhance human capital (Eynon et al., 2018). The growth of human capital can further promote employment opportunities, increase income levels, and provide a practical basis for the improvement of an individual’s subjective social status. On the other hand, individuals actively engage in self-expression on social media (Bailey et al., 2020; Orehek & Human, 2017). By sharing career achievements, family life, and other content, individuals have the opportunity to receive positive feedback within their social networks. Such feedback may psychologically internalize into affirmation of their social value, thus enhancing their subjective social status. Furthermore, the improvement of subjective social status helps to reduce the perception of disadvantage in social comparisons, thereby lowering PRD (Callan et al., 2017). In addition, prior research has shown that subjective social status is significantly negatively associated with various mental disorders and significantly positively associated with mental health levels (Rubin et al., 2016; Scott et al., 2014). Some studies even found that subjective social status is a more effective predictor of mental health than objective social status (Sakurai et al., 2010; Singh-Manoux et al., 2005). These pieces of evidence collectively highlight the potential value of subjective social status in mental health interventions. Compared to interventions that focus solely on material resource provision, enhancing individuals’ subjective social status may be a more feasible approach to alleviating PRD. Future efforts could consider promoting digital health literacy and fostering inclusive online social participation to enhance individuals’ sense of social value in digital environments (Millard et al., 2018; Rivadeneira et al., 2023), thereby improving their subjective social status. These improvements may help buffer the adverse mental health effects of social media use and facilitate a more positive relationship between technological advancement and psychological well-being.

### 4.3. Moderating Role of Belief in a Just World

This study found that belief in a just world not only strengthened the direct negative relationship between social media use and PRD, but also enhanced the positive association between social media use and subjective social status. These findings suggest that individual belief systems play a critical role in shaping social cognition and emotional responses when interpreting social media content. Belief in a just world reflects people’s trust in the logic of “getting what one deserves” (Lerner & Miller, 1978). This belief aligns closely with traditional values in Chinese culture, such as “Heaven rewards the diligent” and “Good deeds will be rewarded”, which reflect expectations of social justice and trust in social order. Individuals with high belief in a just world tend to attribute personal circumstances to internal factors rather than external factors (Furnham, 2003; Hafer & Bègue, 2005; Lerner & Miller, 1978). Specifically, those with high belief in a just world are more likely to perceive the success of others on social media as an achievable goal through personal effort. This interpretation helps boost their confidence in upward mobility, enhance their subjective social status, and alleviate PRD. In contrast, individuals with low belief in a just world are more likely to view the same information as a manifestation of systemic injustice, diminishing their subjective status and reinforcing PRD. Previous studies emphasized that PRD arises from objective social inequality (Greitemeyer & Sagioglou, 2017; Wilkinson & Pickett, 2007). This study further suggests that individuals’ subjective beliefs about social justice are also an important factor in moderating the levels of PRD. Therefore, policies focusing solely on improving external material conditions may be insufficient to effectively reduce PRD. Public health interventions should also aim to strengthen a stable and positive belief system, such as the belief in a just world. Enhancing this belief requires practical measures, including improving transparency in public sectors and ensuring fairness in public services (Alessandro et al., 2021; Cucciniello & Nasi, 2014). Additionally, community engagement programs and mental health education can help individuals develop more accurate perceptions of social fairness. Such actions can better harness social media’s potential to promote psychological well-being among individuals experiencing PRD.

### 4.4. Limitations

Despite providing important insights into the relationship between social media use and PRD among urban residents in China, this study has several limitations. First, it employed a cross-sectional design. Although correlations between variables were identified, causal relationships could not be established. However, as the first study to examine this relationship among urban residents in China, it offers important exploratory insights. Future studies could build on these findings by adopting longitudinal or experimental designs to better clarify causal mechanisms. Second, belief in a just world was measured as a single-dimensional construct, which may overlook the distinct roles of personal belief in a just world and general belief in a just world. Future studies should adopt multidimensional measures to better capture these nuances and improve the accuracy of interaction analyses. Finally, due to constraints in survey implementation, this study focused exclusively on urban residents and did not include rural populations. Given the pronounced urban–rural divide in China, rural residents often face disadvantages in education, employment, and public services. The relationship between social media use and PRD in rural settings may differ significantly. Future research should expand the sample to include both urban and rural populations to allow for more comprehensive and comparative analyses.

### 4.5. Implications

Despite these limitations, the study offers both theoretical and practical contributions. Theoretically, it draws on relative deprivation theory to construct a model linking social media use and PRD, and it is the first to identify a negative association between them among urban residents in China. This finding contrasts with some results from Western contexts and contributes to a broader cross-cultural understanding of the issue. Practically, the results challenge the commonly held view of social media as a mental health risk factor. Evaluating its impact requires consideration of specific cultural contexts and content regulation. Future research could further explore its positive potential for mental health interventions. In addition, subjective social status and belief in a just world are closely related to PRD. Compared to interventions focused solely on material improvements, enhancing these psychological resources may offer a more feasible path. These factors deserve further attention as potential intervention targets.

## 5. Conclusions

This study found that social media use was negatively associated with PRD among urban residents in China. Subjective social status mediated this relationship: social media use was positively associated with subjective social status, while subjective social status was negatively associated with PRD. Moreover, belief in a just world strengthened the direct negative link between social media use and PRD as well as the positive link between social media use and subjective social status. These findings help clarify potential psychological pathways connecting social media use and PRD in the Chinese context and extend the applicability of relative deprivation theory to non-Western settings. The study also highlights the potential positive role of social media in psychological well-being. Future research with more rigorous designs is needed to further test its practical value in mental health interventions.

## Figures and Tables

**Table 1:** Sociodemographic characteristics of participants.

Characteristics	Number of Participants (*n*)	Percentage (%)
**Age**		
18–35	955	38.14
36–59	1321	52.76
≥60	228	9.11
**Gender**		
Male	1198	47.84
Female	1306	52.16
**Marital status**		
Married	1903	76.00
Unmarried	601	24.00
**Education level**		
Primary school or below	163	6.51
Middle school	1293	51.64
College degree or above	1048	41.85
**Annual family income**		
≤CNY 60,000	555	22.16
CNY 60,001–CNY 150,000	886	35.38
≥CNY 150,001	1063	42.45
**Social security status**		
With medical or pension insurance	1910	76.28
Without medical and pension insurance	594	23.72

**Table 2:** Correlation analysis of key variables.

	*M*	*SD*	1	2	3	4
1. Social media use	3.83	0.98	1			
2. Personal relative deprivation	3.85	0.78	−0.257 ***	1		
3. Subjective social status	2.39	0.87	0.153 ***	−0.498 ***	1	
4. Belief in a just world	4.17	1.06	0.138 ***	−0.193 ***	0.224 ***	1

Note: *** *p* < 0.001.

**Table 3:** Mediation analysis.

	Path	*B*	*SE*	*t*	*p*	*LLCI*	*ULCI*
Total effect	SMU → PRD	−0.117	0.018	−6.474	<0.001	−0.152	−0.081
	SMU → SSS	0.072	0.021	3.453	<0.001	0.031	0.113
	SSS → PRD	−0.386	0.016	−24.969	<0.001	−0.417	−0.356
Direct effect	SMU → PRD	−0.089	0.016	−5.498	<0.001	−0.121	−0.057
Indirect effect	SMU → SSS → PRD	−0.028	0.008			−0.045	−0.012

Note: SMU = social media use, PRD = personal relative deprivation, SSS = subjective social status. SE = standard error, LLCI = lower limit 95% confidence interval, ULCI = upper limit 95% confidence interval.

**Table 4:** Moderated mediation analysis.

Variables	PRD				SSS			
	*B*	*SE*	*t*	*p*	*B*	*SE*	*t*	*p*
SMU	−0.086	0.016	−5.328	<0.001	0.058	0.021	2.805	<0.01
BJW	−0.048	0.013	−3.774	<0.001	0.160	0.016	10.040	<0.001
SSS	−0.374	0.016	−23.771	<0.001				
SMU × BJW	−0.025	0.012	−2.074	<0.05	0.047	0.016	3.016	<0.01
Age	−0.002	0.001	−1.265	0.206	0.008	0.002	5.307	<0.001
Gender	−0.108	0.026	−4.195	<0.001	−0.110	0.033	−3.337	<0.001
Marital status	−0.060	0.032	−1.853	0.064	0.023	0.041	0.554	0.580
Education level	−0.080	0.015	−5.322	<0.001	0.115	0.019	6.066	<0.001
Annual family income	−0.096	0.013	−7.342	<0.001	0.118	0.017	7.178	<0.001
Social security status	−0.040	0.031	−1.300	0.194	0.057	0.039	1.435	0.152
*R* ^2^	0.322				0.121			
*F*	118.254				38.288			

Note: SMU = social media use, PRD = personal relative deprivation, SSS = subjective social status, BJW = belief in a just world. SE = standard error.

## Data Availability

The raw data supporting the conclusions of this article will be made available by the authors on request.

## Supplementary Materials

The following supporting information can be downloaded at: https://www.mdpi.com/article/10.3390/bs15070962/s1.

## Abbreviations

The following abbreviations are used in this manuscript:

PRD	Personal relative deprivation
SMU	Social media use
SSS	Subjective social status
BJW	Belief in a just world
