# Supplementary material for: Social Media Use and Personal Relative Deprivation Among Urban Residents in China: A Moderated Mediation Model
Source: Behav Sci (Basel). 2025 Jul 16;15(7):962. doi: 10.3390/bs15070962 (PMC12292491; doi:10.3390/bs15070962)
Supplement: Supplementary file 1 [file behavsci-15-00962-s001.zip › behavsci-3699470-supplementary.pdf]

behavioral sciences

Table S1. Mediation analysis.

	Path	β	*SE*	*t*	*p*	*LLCI*	*ULCI*
Total effect	SMU→PRD	−0.147	0.023	−6.474	<0.001	−0.192	−0.103
	SMU→SSS	0.081	0.024	3.453	<0.001	0.035	0.127
	SSS→PRD	−0.432	0.017	−24.969	<0.001	−0.466	−0.398
Direct effect	SMU→PRD	−0.112	0.020	−5.498	<0.001	−0.152	−0.072
Indirect effect	SMU→SSS→PRD	−0.035	0.011			−0.056	−0.015

Note. All coefficients are standardized. SMU = Social media use, PRD = Personal relative deprivation, SSS = Subjective social status. SE = Standard error, LLCI = Lower limit 95% confidence interval, ULCI = Upper limit 95% confidence interval.

Table S2. Moderated mediation analysis.

Variables	PRD				SSS			
	β	*SE*	*t*	*p*	β	*SE*	*t*	*p*
SMU	−0.109	0.020	−5.328	<0.001	0.065	0.023	2.805	<0.01
BJW	−0.066	0.017	−3.774	<0.001	0.195	0.019	10.040	<0.001
SSS	−0.418	0.018	−23.771	<0.001				
SMU × BJW	−0.034	0.016	−2.074	<0.05	0.056	0.019	3.016	<0.01
Age	−0.025	0.020	−1.265	0.206	0.118	0.022	5.307	<0.001
Gender	−0.140	0.033	−4.195	<0.001	−0.126	0.038	−3.337	<0.001
Marital status	−0.077	0.042	−1.853	0.064	0.026	0.047	0.554	0.580
Education level	−0.106	0.020	−5.322	<0.001	0.136	0.022	6.066	<0.001
Annual family income	−0.132	0.018	−7.342	<0.001	0.145	0.020	7.178	<0.001
Social security status	−0.052	0.040	−1.300	0.194	0.065	0.045	1.435	0.152
R^2	0.322				0.121			
F	118.254				38.288			

Note. All coefficients are standardized. SMU = Social media use, PRD = Personal relative deprivation, SSS = Subjective social status, BJW = Belief in a just world. SE = Standard error.
